# Modeling pediatric brain tumors with human stem cells

**DOI:** 10.3389/fncel.2026.1720855

**Published:** 2026-02-11

**Authors:** Noah Burket, Titto Augustine, Jignesh Tailor

**Affiliations:** 1 Department of Neurological Surgery, Indiana University School of Medicine, Indianapolis, IN, United States; 2 Herman B Wells Center for Pediatric Research, Indianapolis, IN, United States; 3 IU Simon Comprehensive Cancer Center, Indianapolis, IN, United States

**Keywords:** 3D models, central nervous system, embryonic stem cell, glioma, induced pluriopotent stem cells, neuroepithelial stem cells, pediatric brain tumor, progenitor cells

## Abstract

With recent advances in stem cell technology, there has been an expansion of human stem and progenitor cell models of pediatric brain tumors, including use of human pluripotent and embryonic stem cells both in organoid cultures and following xenotransplantation in mice. In this review, we discuss the current approaches to modelling pediatric brain cancers using stem cells. While brain tumors describe a broad set of disease entities, we focus on glioma, medulloblastoma and ependymoma, as these are not only the most common malignant brain tumor types but also have the most stem cell models currently available. We examine human stem cell-based modeling approaches and discuss the biological questions that are being addressed using these state-of-the-art tools. Specifically, we focus on the unique advantage of using these cells to understand the functional consequences of gene mutations and their downstream growth-promoting pathways within the cell in a human context. These approaches are needed to ascertain the key players that are functionally relevant in the initiation and propagation of these tumors at the gene and protein level and to identify new drug targets. Moreover, human stem cell-based modeling approaches may complement studies in genetically engineered mouse models to address fundamental questions in tumor biology, particularly the early stages of tumorigenesis.

## Introduction

Central nervous system (CNS) tumors present a significant clinical challenge due to their complex nature, biological variability, and profound impact on patients. CNS tumors are the most common cause of cancer death in children and the second most common cause of cancer deaths for adolescents and young adults, second only to breast cancer (Price et al., 2024). Understanding the epidemiology and clinical behavior of these tumors is crucial for developing targeted therapies and improving patient outcomes. Pediatric CNS tumors differ in their pathology, location, and prognosis (d’Amati et al., 2024). The most common tumors include high- and low-grade gliomas, medulloblastoma, and ependymomas; however, other rarer pediatric neoplasms exist (d’Amati et al., 2024). Their heterogeneity reflects the extraordinary cellular diversity of the CNS, where lineage specification and differentiation are tightly regulated by spatiotemporal developmental programs (d’Amati et al., 2024). Defining how genetic and epigenetic mutations alter these programs to drive tumorigenesis is critical for developing effective therapies. Moreover, inherited tumor predisposition syndromes, such as neurofibromatosis (type 1 & 2), Li-Fraumeni, and Gorlin syndrome, further increase the risk of not only development of CNS tumors but are also associated with risk of recurrence and poor outcomes for patients (Patil et al., 2022). These patients are typically enrolled in screening programs in specialized clinics, yet, few treatment options exist to stop progression of disease (Hansford et al., 2024).

Progress within the field has been constrained by the limitations of traditional experimental models. While mouse models and tumor-derived cell lines have provided key insight into oncogenic signaling, cell-of-origin, and therapeutic vulnerabilities, they fail to capture essential aspects of human brain tumor biology (Huszthy et al., 2012). Species-specific differences in development, the inability to model early human neurogenesis, and the divergence of tumor cell lines from their parental state have limited the impact of these approaches (Zhao and Bhattacharyya, 2018). Further, most available models do not faithfully reproduce the microenvironmental context, immune composition, or spatial architecture that shape tumor growth and treatment response in human patients (de and Maina, 2010).

Stem and progenitor cell-based models have emerged as powerful tools for studying brain tumor biology and mechanisms of drug response and resistance (Dave and Tailor, 2024). Unlike the use of traditional cell lines and animal models, stem cell-derived models can better recapitulate human tumor heterogeneity, epigenetic dynamics, and patient-specific genetic alterations (Qian et al., 2019). By leveraging these models, researchers can systematically evaluate genetic dependencies, tumor vulnerabilities, and novel therapeutic targets, improving the development of precision medicine approaches for CNS tumor treatment (Chehelgerdi et al., 2023). In this review, we summarize recent advances in human stem cell-based modeling of CNS tumors and evaluate their potential to improve our understanding of tumor biology and therapeutic options.

## Overview of brain tumor models

### Traditional brain tumor models and their limitations

The earliest preclinical models for brain tumors were created by introducing carcinogenic materials, such as methylcholanthrene or N-nitrosourea, into *in vivo* and *in vitro* systems (Dobson and Gopalakrishnan, 2018). These early approaches to creating tumors in animal models were incredibly impactful for the neuro-oncology field; however, the carcinogen-induced systems did not always create tumors and could result in tumors that no longer resembled the original tumor after multiple passages (Peterson et al., 1994). Around this same time, other groups began using oncogenic viruses to induce tumors in animal models, which were more reliable at creating tumors than carcinogenic techniques but came with the same limitation of varied histopathology and tumor characteristics (Dobson and Gopalakrishnan, 2018).

Nearly a decade later, investigators created the first transplantation models for brain tumor research (Dobson and Gopalakrishnan, 2018). These models primarily fall into two categories: syngeneic and xenograft models. Syngeneic models utilize cells or tissue from one animal to create a tumor in a different animal from the same species. Early on, groups took carcinogen- or viral-induced mouse brain tumor tissue and either directly implanted it into other mice or first grew it in culture before implantation (indirect implantation) (Dobson and Gopalakrishnan, 2018). This increased the feasibility of larger studies investigating drugs and chemotherapy agents to treat tumors that arise *in vivo*. Today, the use of genome editing tools, such as CRISPR and PiggyBac systems, have improved syngeneic mouse models, but there has been a shift toward xenograft models due to their better recapitulation of human brain tumors (du Chatinier et al., 2022; Zhong et al., 2020). Unlike syngeneic models, xenograft models involve the implantation of cells or tissue from a donor that is a different species than the host. Xenografts are notably effective in modeling brain tumors, as they can retain the genetic, epigenetic, and histopathological characteristics of the original patient tumor (Joo et al., 2013; Vaubel et al., 2020). The primary drawback for these models is that they require a host that is immunocompromised to allow for the implanted tissue to grow and form tumors (Mann et al., 2025; He et al., 2021). One of the most common animals used for these studies are athymic or nonobese diabetic/severe combined immunodeficiency mice, which lack mature immune cells, preventing the host from mounting a defense against the engraftment. While this improves engraftment in the host animal, it prohibits the study of the complex interplay between brain tumors and the immune system in the tumor microenvironment (Mann et al., 2025). More recently, humanized mouse models have been developed which either engraft human hematopoietic stem cells or use genome editing tools in immunodeficient mice so that they express human immune system components (Yang et al., 2024). Once created, patient tumor tissue can be implanted into these humanized mice, allowing researchers to study interactions between the immune system and tumor cells in a system that is closer to what is seen in humans (Srivastava et al., 2023). Yet, all other cells and tissues outside of the patient tumor and engineered immune cells retain their mouse origin, limiting the model’s ability to truly capture unique human tumor microenvironments. Furthermore, no humanized mouse models currently exist specifically for pediatric brain tumors.

**Figure 1:**
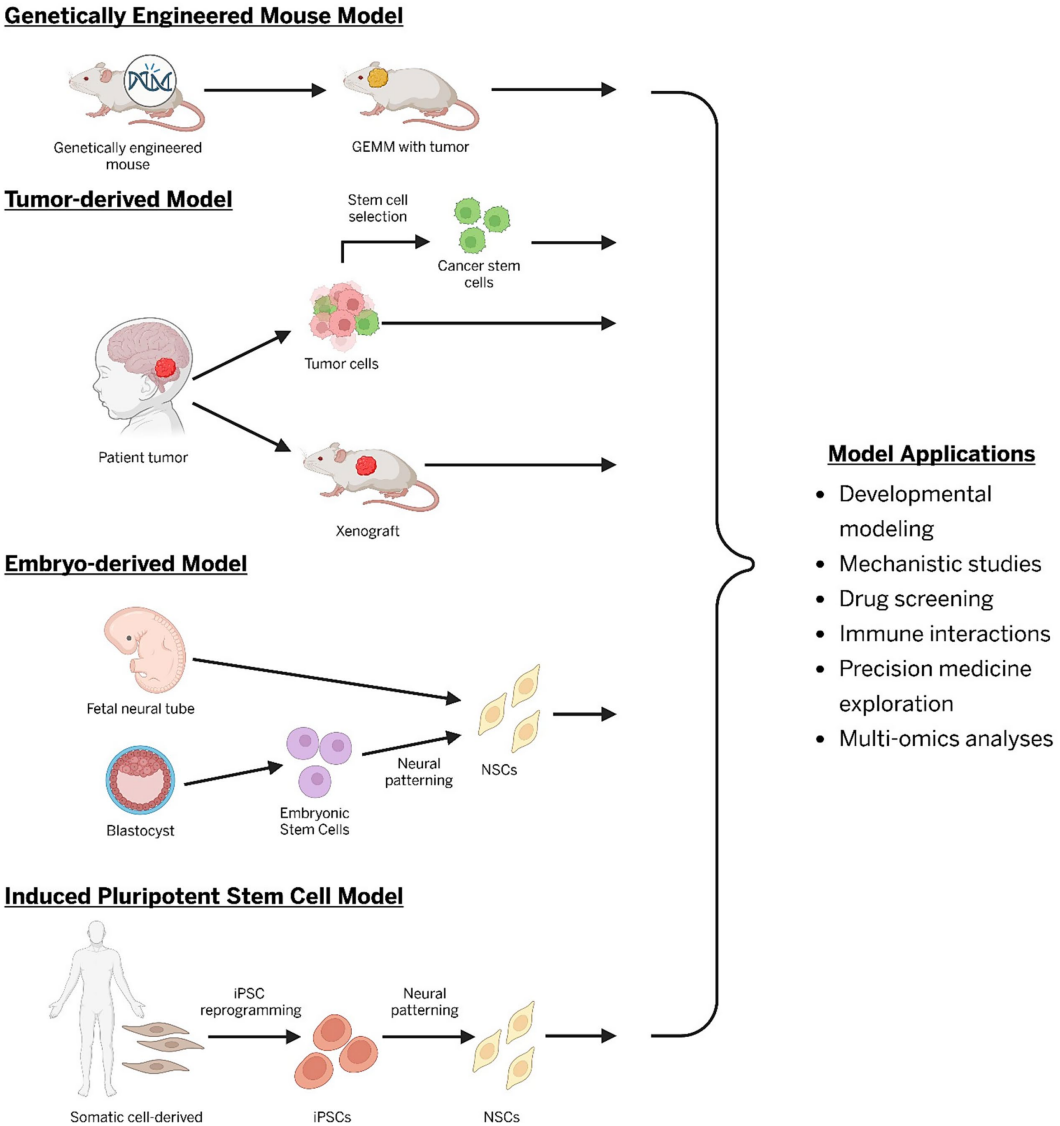
Types of common preclinical models for central nervous system tumors (GEMM, genetically engineered mouse model; NSCs, neural stem cells; iPSC, induced pluripotent stem cells).

Transgenic models and genetically engineered mouse models (GEMMs) have allowed researchers to study the development and treatment of brain tumors due to the increasing ease of genetic manipulations (Figure 1) (Antonica et al., 2022; Burket, 2025a). These models enable the study of the roles of precise oncogenic mutations in tumorigenesis and downstream pathways. Inducible systems further allow for interrogation of cell type-specific and temporal-dependent effects of genetic alteration (Larson et al., 2019; Alcantara Llaguno et al., 2009; Alcantara Llaguno et al., 2019). Despite these advantages, GEMMs and transgenic models are limited in their ability to model brain tumors that depend on human-specific biology. Although mouse and human brains have many similarities, mice lack key structural and cellular features that may be necessary for tumor development. For example, differences include expanded cortical layers (especially layers II and III) and a proportionally larger visual cortex in humans (Kanari et al., 2025; Lancaster, 2024). Additionally, mice lack an expanded outer subventricular zone and specialized neural cell types, such as rosehip neurons (Dehay et al., 2015; Boldog et al., 2018). As a result, some brain tumors may be challenging to model without xenografts or human stem cell-based platforms. Furthermore, while these models can recapitulate key genetic drivers, they often fail to fully capture the tumor heterogeneity and epigenetic landscape seen in human pediatric brain tumors (Huszthy et al., 2012; Filbin and Monje, 2019; Huang et al., 2019). The long generation times and high costs associated with developing and maintaining these mouse models further limit their applicability and feasibility for certain studies (Sun et al., 2021).

Lastly, conventional tumor-derived and immortalized cell lines have been essential for mechanistic studies and high-throughput screening due to their ease of establishment, scalability, and reproducibility (Figure 1) (Burket, 2025a; Desai et al., 2023; Mirabelli et al., 2019). Yet, these models fail to represent intratumoral heterogeneity, exhibit genomic instability, and typically rely on more mature cell states, limiting their ability to truly model tumor initiation and developmental effects caused by mutations (Dave and Tailor, 2024; Arbatskiy et al., 2024; Peng et al., 2025; Salvadores et al., 2020). While foundational for elucidating brain tumor biology, traditional cell lines fail to capture the full complexity of human tumors. These limitations underscore the need for complementary approaches with human stem cell-based systems, which provide a more physiologically relevant platform that enables modeling of many tumors, including those that grow poorly in culture.

### Advantages of human stem cell modeling

The term stem cell is defined as cells that can both self-renew, and maintain the capacity to produce daughter cells that differentiate to defined lineages (Smith, 2006). Researchers initially began propagating human progenitor cells *in vitro* through cell line immortalization or in neurospheres. These cells would often differentiate spontaneously, hampering long term expansion through self-renewal. Embryonic stem cells (ESC) were first isolated and propagated in culture from the human blastocyst in 1998 (Thomson et al., 1998). Around this same time, neural stem cells (NSCs) were being characterized in the embryonic and postnatal brain, accelerating this area of research and providing more insight into early brain development (Reynolds and Weiss, 1992; Brüstle et al., 1997; Breunig et al., 2011). Advances in stem cell technology led to the first human NSC line that was propagated in culture long-term from the embryonic human brain in a 2D mono-layer (Sun et al., 2008). In 2006, Shinya Yamanaka successfully created the first induced pluripotent stem cells (iPSCs) by using four transcription factors to reprogram mouse and human fibroblasts (Takahashi and Yamanaka, 2006; Takahashi et al., 2007). These groundbreaking investigations fueled additional studies that utilized iPSCs to create cells from all three germinal layers, including the ectoderm, from which neural stem cells arise to form the CNS (Evseenko et al., 2010; Teo et al., 2011; Chambers et al., 2009; Hu et al., 2010). Together, the derivation of NSC lines from the ESCs and iPSCs has revolutionized the field of stem cell research and provided the foundation for the creation of new preclinical models that more closely mimic human development and biological processes (Figure 1) (Burket, 2025a).

Neuroepithelial stem cells are considered the foundational stem cells of the CNS during development, giving rise to radial glia (RG) cells, which can further differentiate into neurons and glia (Miranda-Negrón and García-Arrarás, 2022; Eze et al., 2021). During human neurodevelopment, these stem and progenitor cells respond to different molecular and environmental cues that govern not only which mature neural cells they will ultimately give rise to, but also their regional identity (Hitoshi et al., 2002; Imaizumi and Okano, 2021). Deviations in this process, due to anything from environmental toxins to genetic mutations, may lead to abnormal development and pathological consequences, including pediatric brain tumorigenesis (Baker et al., 2016; Wylie and Short, 2023; Farouk Sait et al., 2021; Jacques et al., 2010). Neuroepithelial progenitors can be captured as neuroepithelial stem (NES) cells *in vitro*, and these stem cells can be leveraged to recapitulate these early developmental alterations that may initiate brain tumor development (Tailor et al., 2013).

NES cell models have the distinct advantage of recapitulating the origin and evolution of brain tumors (Heinzelmann et al., 2024). Furthermore, stem cell lines will self-renew indefinitely, providing opportunity to recapitulate genetic events in low-grade tumors, which can be notoriously difficult to model using tumor-derived cell lines (Anastasaki et al., 2022). Additionally, the epigenetic landscape of human stem cell derived brain tumor models may be closer to patient derived tumor than mouse counterparts (Huang et al., 2019). Previous reports have noted specific cell types/processes are present in humans that are absent in mice, as well as lower accuracy of mouse models in predicting therapeutic responses for brain tumors (Mann et al., 2025; Haldipur et al., 2019; Hicks et al., 2021). This may be due to differences in genetic and epigenetic structure or in the brain microenvironment, but nonetheless highlights the utility and necessity of using human cells and tissue to study human-specific tumors.

**Figure 2:**
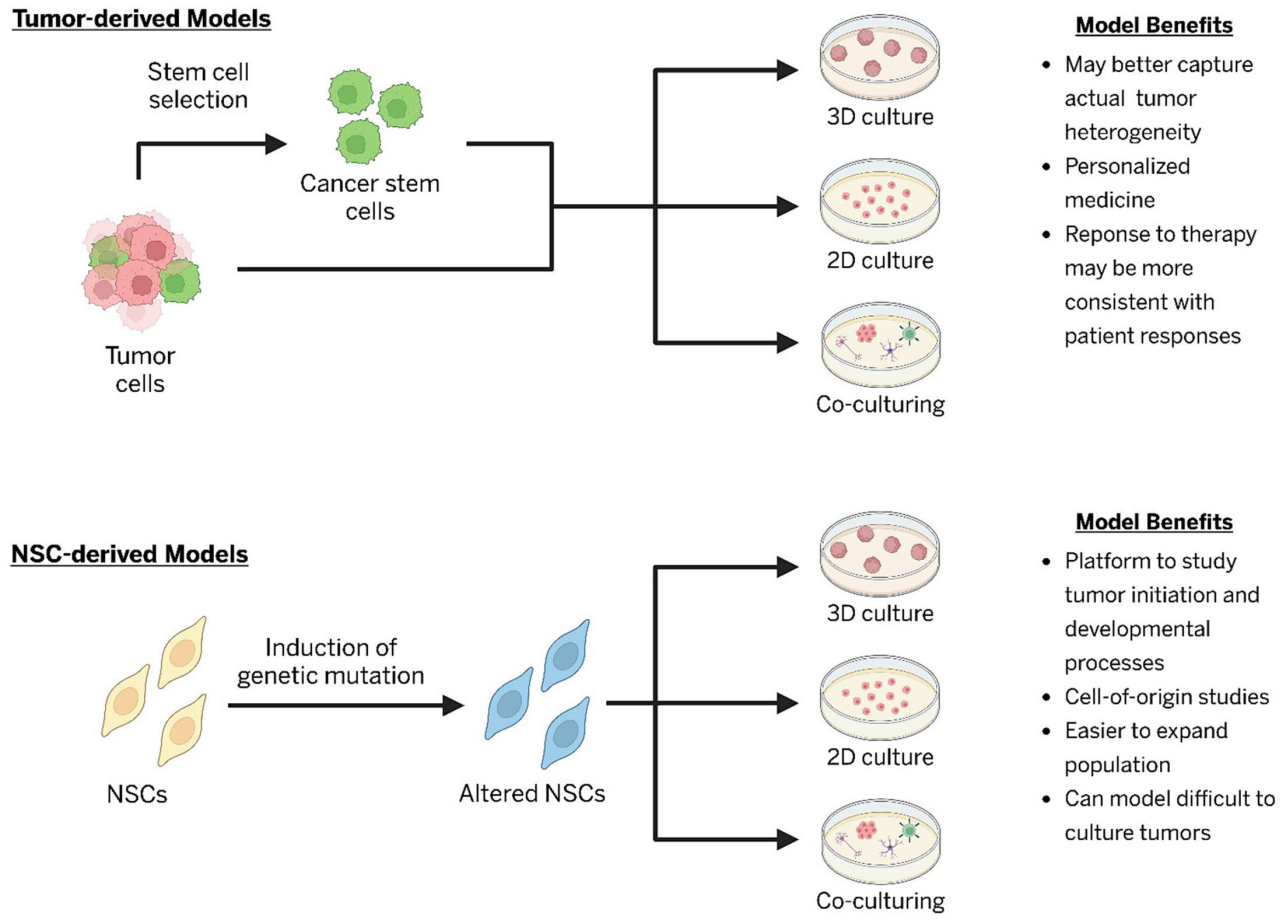
Human cell-based models and their common applications and benefits (NSCs, neural stem cells).

NSCs may also be related to cancer stem cells (CSCs), which are a subpopulation of tumor-initiating cells within a tumor that are thought to promote recurrence and treatment resistance (Singh et al., 2004). CSCs can be isolated from multiple pediatric brain tumors, including high-grade gliomas and medulloblastomas, and have been isolated as tumor-derived cell lines (Lin et al., 2023). These cells can be maintained in culture and implanted in mice as xenografts, recapitulating markers and behaviors seen in the parental tumors (Singh et al., 2004; Haynes et al., 2019; Hemmati et al., 2003). These cells provide an excellent platform for studying many important factors involved in human brain tumorigenesis. However, CSCs may not survive *ex vivo*, can lose stem cell properties after several passages, and are occasionally difficult to derive from low-grade or indolent tumors (Mann et al., 2025; Wenger et al., 2017). Importantly, these cells may not be representative of early tumorigenesis, limiting their use in cell-of-origin studies (Bamodu et al., 2024). To circumvent this issue, normal neural stem cells derived from iPSCs and ESCs can be engineered to model the functional effects of mutations seen in the CSCs but at earlier cell stages, such as at the NES cell or RG cell timepoint (Figure 2) (Kong, 2012; Burket, 2025b).

### Three-dimensional organoid systems

Three-dimensional (3D) culture techniques have allowed for transformative advances in tumor modeling within the field of neuro-oncology. Several different types of models exist, including tumoroid, iPSC-derived organoids, and tumor-organoid cultures. Tumoroid models rely only on patient tumor cells to create 3D structures that may better represent tumors *in vivo* by replicating complex cell–cell interactions and tumor microenvironments (Kalla et al., 2025). Stem cell-derived brain organoids on the other hand use human iPSC or ESC cells to create 3D structures composed of various “normal” cells that are found within the brain (Shayestehfar et al., 2025). Some models opt for an unguided differentiation protocol, allowing cells that form the 3D structure to differentiate into various cell types from different regions within the brain (Xiang and Park, 2024). Improving on this, researchers have also used patterning protocols to create organoids that represent different regions of the brain, allowing more precise control on the cell types and regionalization that may impact biological findings (Renner et al., 2017; Khamis et al., 2023). Genetic alterations introduced into these systems can drive neoplastic transformation within a human developmental context, capturing aspects of tumor initiation, growth, and heterogeneity absent in 2D cultures (Bian et al., 2018). Tumor-organoid cultures have also been generated by co-culturing patient tumor tissue with iPSCs, preserving the histology, genetic mutations, and drug responses of the parental tumors (Peng et al., 2025; Azzarelli et al., 2021). Drug screening on these organoids revealed heterogeneous responses consistent with patient outcomes, highlighting their potential as predictive platforms for personalized therapy (Peng et al., 2025). Importantly, organoid models allow investigation of tumor-microenvironment interactions that shape the growth and sensitivity to potential therapeutics (Peng et al., 2025; Azzarelli et al., 2021). Non-neural cells, such as microglia and stromal cells, are known to impact brain tumor growth and response to treatment (Coniglio et al., 2012; Herrera-Perez et al., 2018; Clavreul and Menei, 2020; Tripathy et al., 2024). Microglia or vascular cell-containing organoids have recently been generated through either co-culture methods or separate differentiation protocols to study different nervous system pathologies (Cakir et al., 2019; Cakir et al., 2022; Xu et al., 2021; Wenzel et al., 2024; Dao et al., 2024; Rittenhouse et al., 2025). These platforms better mimic the complex interactions within the human brain compared to 2D cultures or murine models and may allow researchers to better understand human brain tumor development, progression, and treatment, underscoring the unique strengths of organoid-based co-culturing approaches (Figure 2) (Burket, 2025b; Raguin et al., 2024).

These 3D models offer a versatile and physiologically relevant option for studying brain tumor biology. By maintaining microenvironmental features while also enabling genetic manipulations and modeling human-specific development trajectories, these systems overcome many limitations of conventional tumor models, including other human stem cell-based 2D models. As protocols are refined and expanded to include more complex developmental structures and processes, these models will become an attractive and needed tool in the field of neuro-oncology.

## Application of human stem cell models for pediatric brain tumors

The diversity of brain tumors highlights the need for specific models to recreate their complex developmental origins. Stem cell-based approaches offer a unique opportunity to faithfully recapitulate this within the appropriate lineage and temporal contexts, providing a more robust understanding of how genetic mutations reprogram developmental events to drive tumorigenesis. Below, we summarize many of the approaches for various human stem cell-derived pediatric brain tumor models and their role in advancing the field of neuro-oncology (Table 1).

**Table 1:** Information on primary drivers, drivers engineered in current models, proposed cell lineage, and types of models created for pediatric brain tumors.

Tumor model	Primary drivers	Mutations in current stem cell models	Proposed cellular lineage	Stem cell model type				References
				iPSC 2D	ESC 2D	Stem cell organoid	Tumor-organoid	
High-grade glioma	DMG: *H3K27M, EZHIP, EGFR* DHG: *H3G34R, H3G34V* pHGG H3/IDH WT: *PDGFRA, EGFR, TERT, MYCN* Infantile Hemispheric Glioma: *ALK, NTRK, ROS1, MET*	DMG: *H3.3K27M, PDGFRA, TP53* DHG: *H3.3G34R, TP53, PDGFRA, ATRX, MYCN*	DMG: Brainstem neural progenitors DHG: Forebrain neural progenitors	✔	✔	✔	✔	2,89–96
Low-grade glioma	KIAA1549: BRAF, BRAF V600E, *NF1*, *FGFR1*, *MYB-QKI*, *MYBL1*, *PRKCA*	BRAF V600E, IDH1 R132H, *ATRX*, *TP53*, *NF1*, KIAA1549: BRAF, *CDK4*, *EGFR*, *PDGFRA*	Neural progenitors, glial progenitors	✔	✔	✘	✘	2,60,97,99–101
Medulloblastoma	WNT MB: *CTNNB1, APC, DDX3X, KMT2D* SHH MB: *PTCH1, TP53, KMT2D, DDX3X, MYCN, BCOR, LDB1, GLI2, SUFU, PALB2, BRCA2* G3 MB: *MYC, OTX2, SMARCA4, NOTCH, TGF-beta, PALB2, BRCA2* G4 MB: *MYCN, CDKN6, SNCAIP, PALB2, BRCA2*	SHH MB: MYCN, DDX3X, GSE1, KDM3B, PTCH1 G3 MB: MYC, TP53, GFI1, OTX2	SHH MB: Rhombic lip granule neuron progenitors G3 MB: Rhombic lip neural progenitors	✔	✔	✔	✘	2,102–108
Ependymoma	Supratentorial: ZFTA: RELA, YAP1: MAMLD1 Posterior Fossa: *EZHIP* Spinal: *MYCN, NF2*	Primary tumors with various mutations reported	Radial glia cells/Progenitors within the ependymal cell lineage	✘	✘	✘	✔	2,109–121
Atypical teratoid/rhabdoid tumors	*SMARCB1, SMARCA4*	*SMARCB1, TP53*	Neural progenitors, Pluripotent fetal progenitors	✔	✘	✔	✘	2,122–124
Craniopharyngioma	*CTNNB1*	-	Precursor cells from Rathke’s pouch epithelium	✘	✘	✘	✘	128–130
Pineoblastoma	*RB1, DICER1, DROSHA, DGCR8, MYC, MYCN, FOXR2*	-	Pinealocytes	✘	✘	✘	✘	131–134
Embryonal tumors with multilayered rosettes	*C19MC, DICER1*	*C19MC*	Radial glia cells	✘	✘	✘	✔	2,135,136
Choroid plexus tumors	*TP53, MYC*	-	Choroid plexus epithelial cells	✘	✘	✘	✘	2,137–139,141–148
Intracranial germ cell tumors	KIT/RAS pathway, PI3K/mTOR pathway	-	Pluripotent embryonic cells	✘	✘	✘	✘	149–152

### High-grade gliomas

Pediatric high-grade gliomas (pHGGs) are comprised of several groups of molecularly distinct tumors, including but not limited to diffuse midline glioma, H3 K27-altered; diffuse hemispheric glioma, H3 G34-mutant; diffuse pediatric-type high-grade glioma, H3-wildtype and IDH-wildtype; and infant-type hemispheric glioma (d’Amati et al., 2024). These pediatric tumors often differ significantly from adult high-grade gliomas in anatomical location, histopathology, and prognosis, necessitating the development of human stem cell-based models that accurately recapitulate their specific biology and behavior.

ESC-derived models were some of the first human stem cell systems to model pHGG tumorigenesis. Introduction of *PDGFRA*, *p53*, and H3.3K27M mutations produced a triple-mutant diffuse midline glioma (DMG) model with enhanced cell survival, migration, invasion, proliferation, and radioresistance (Funato et al., 2014). Implantation of these cells into mice produced tumors with histological and molecular features resembling low-grade DMGs, including upregulation of neural progenitor genes (Funato et al., 2014). Subsequent work using ESC-derived neural progenitors harboring G34R, *ATRX*, and p53 mutations to model H3.3G34R glioma (Funato et al., 2021). The addition of *MYCN* overexpression and ventral forebrain patterning – but not hindbrain – disrupted neuronal differentiation and formed tumors *in vivo*, demonstrating the preferential role of regional identity in tumorigenesis (Funato et al., 2021). Complimentary studies revealed that K27M mutations are primarily tumorigenic in brainstem progenitors, whereas G34R mutations promote proliferation in forebrain progenitors when combined with *PDGFRA* amplification and *TP53* loss (Bressan et al., 2021). This mirrors typical anatomic restriction of these tumors seen in patients (d’Amati et al., 2024). Mechanistic experiments revealed that K27M- and G34R-driven tumorigenesis appear to operate based on distinct epigenetic processes, leading to increased chromatin accessibility and disrupted neural progenitor differentiation (Bressan et al., 2021; Brien et al., 2021). Together, these studies highlight how mutations within region-specific cells drive pHGG initiation.

iPSC-derived models have further expanded these findings by enabling experimentation with driver mutations across different neural lineages. Haag et al. generated H3.1- and H3.3K27M mutations in multiple neural lineages and found that only neural stem cells with H3.3K27M mutations displayed oncogenic phenotypes, particularly when combined with *TP53* knockdown (Haag et al., 2021). Tumors that formed from these cells were aggressive, showed leptomeningeal spread, and had DNA methylation and transcriptomic profiles similar to K27M mutant DMG (Haag et al., 2021). In a preprint, Skinner et al. introduced *TP53*, H3.3K27M, and *PDGFRA* mutations in iPSC-derived neural progenitor cells (Haag et al., 2021). Cells with H3.3K27M and *TP53* mutations formed tumors in mice that showed similar histology to patient DMGs (Skinner et al., 2023). This triple mutant cell line was an improvement over the model created by Haag et al. due to the addition of PDGFRA overexpression, creating tumors which were much more infiltrative than those with H3.3K27M and p53 loss alone, as well as having a metabolic signature highly similar to H3.3K27M DMGs (Skinner et al., 2023).

Three-dimensional human stem cell-based systems have incorporated aspects of the surrounding brain microenvironment. Immune-competent tumor-organoids combining DMG spheroids with microglia, myeloid cells, and ESC-derived neural progenitors revealed microglia-enhanced invasion and microenvironmental components that affect tumor infiltration and immune responses (Sarnow et al., 2025). Additional studies created patient-derived tumoroids from H3K27M DMG samples that exhibited invasive behavior and oligodendrocyte progenitor-like transcriptional signatures (Bruschi et al., 2023). These models are important next steps in creating more representative models to study pHGG *in vitro* and could be useful for drug screens to identify novel therapies for treating these tumors.

Collectively, ESC-, iPSC-, and organoid-based models have enabled the study of the effects of genetic mutations within specific developmental lineages while capturing human-specific tumor features that are difficult to model in mice or tumor-derived cultures alone. These systems continue to be essential for uncovering the role of molecular mutations in pHGG tumorigenesis, especially when considering the importance of key developmental phases in which these mutations must occur. Future studies may benefit from using advanced modeling techniques, such as co-culturing with endothelial cells and other immune or neural cells to more accurately model human tumors. Further, there are opportunities to use human NSC models to investigate the other pHGG subtypes.

### Low-grade gliomas

Low-grade gliomas (LGGs) represent another diverse group of pediatric CNS tumors, accounting for approximately 30% of cases (d’Amati et al., 2024). These tumors are now grouped into three primary types: pediatric-type diffuse low-grade gliomas, circumscribed astrocytic gliomas, and glioneuronal and neuronal tumors (d’Amati et al., 2024). Most harbor MAPK pathway alterations that increase proliferation, migration, and survival (Lindsay et al., 2023). However, LGGs can be challenging to model using patient tissue due to low proliferative rates, limited surgical accessibility, and oncogene-induced-senescence (Yvone and Breunig, 2024). This makes modeling with human stem cell-based systems a superior alternative to mouse models and primary tumor-derived cell cultures for their availability, ease of specific genetic editing, and scalability.

Early work using fetal-derived neural progenitors showed that introducing BRAF V600E mutations activated downstream ERK signaling and cell colony formation but failed to increase cell proliferation (Raabe et al., 2011). Continued passaging of this line lead to oncogene-induced-sensence (Raabe et al., 2011). This recapitulated a key limitation of primary LGG cultures and demonstrated how initiating mutations may be insufficient in this model without additional genetic edits.

Human ESC-derived models have also been used to model IDH1 mutations in LGG tumorigenesis. NSCs with IDH1 R132H mutations, with or without p53 and ATRX loss, mirrored the transcriptomic and methylation profiles of patient tumors (Modrek et al., 2017). IDH1 mutations alone or with ATRX loss reduced cell viability via apoptosis, which was rescued with p53 loss (Modrek et al., 2017). Stalled differentiation was noted in the IDH1-mutant and triple mutant models through increased DNA methylation of CTCF sites mediated by downregulation of *SOX2* (Modrek et al., 2017). Ectopic SOX2 expression restored differentiation in these cells, supporting that IDH1-mutant LGGs may arise from differentiation arrest in neural progenitors (Modrek et al., 2017). This process is difficult to capture in tumor-derived cell lines and xenografts given their inability to model early developmental events in tumorigenesis.

iPSC-derived models have further advanced LGG modeling by enabling interrogation of lineage preferences. Anastasaki et al. generated iPSCs harboring *NF1* loss or *KIAA1549: BRAF* fusion mutations and differentiated them into multiple neural lineages (Anastasaki et al., 2022). Only neural progenitor and oligodendrocyte progenitor cells, but not mature astrocytes, carrying these mutations formed tumors with histopathological features of LGG when implanted into the hindbrain of *Rag1*−/− mice (Anastasaki et al., 2022). Interestingly, Cxcl10 secreted by astrocytes suppressed LGG engraftment, emphasizing a critical role for the tumor microenvironment in modulating tumor growth (Anastasaki et al., 2022). Treatment with MEK inhibitors increased apoptosis and reduced proliferation *in vitro* and *in vivo*, validating this model system for therapeutic testing (Anastasaki et al., 2022). Further insights into lineage specificity effects were discovered after engineering human prenatal NSCs, glial progenitors, and oligodendrocyte precursor cells with p53 R175H mutations and NF1 loss (Liu et al., 2025). While all cell lines formed tumors *in vivo*, each exhibited distinct lineage outcomes: NSC-derived tumors generated neuronal-like cells, whereas glial progenitors skewed toward astrocytic lineages and oligodendrocyte precursors favored oligodendrocyte differentiation (Liu et al., 2025). Introduction of additional mutations in NSCs, including *CDK4*, *EGFR*, or *PDGFRA*, altered lineage outcomes (Liu et al., 2025). Together, these studies demonstrate the power of stem cell-based models in capturing the relationship between specific genetic events and developmental states in shaping LGG phenotypes.

Despite these advances, 3D LGG models have not yet been established. Given the success of organoid and tumoroid systems for other brain tumors, adaptation of this type of model represents an important future direction. These models could provide a more physiologically relevant context to study tumor progression, differentiation, and microenvironmental interactions in pediatric LGGs. Nevertheless, LGG studies have clearly benefited from human stem cell modeling.

### Medulloblastoma

Medulloblastoma, the second most common pediatric brain tumor, is reclassified into four major groups: WNT, SHH, Group 3, and Group 4 (d’Amati et al., 2024). Human stem cell-based models have been essential in recapitulating these tumor subtypes, particularly SHH and Group 3 medulloblastomas, and elucidating mechanisms of tumorigenesis, cell-of-origin, and therapeutic vulnerabilities.

ESC-derived NSCs with *MYC* overexpression alone or in combination with constitutively active *AKT*, dominant-negative *TP53*, and human telomerase generated anaplastic tumors *in vivo* (Hanaford et al., 2016). NSCs with all four mutations exhibited histologic and transcriptomic features that closely matched Group 3 MB at the transcriptomic and histologic levels (Hanaford et al., 2016). Parallel work using iPSC-derived ATOH1-positive neuronal progenitors with *MYC* overexpression and p53 loss similarly produced Group 3-like tumors *in vivo* (Xue et al., 2021). Pharmacological treatment in both model systems resulted in reduced cell survival, hinting at potential therapies for this tumor subtype (Hanaford et al., 2016; Xue et al., 2021). Together, these studies demonstrate that both iPSC- and ESC-based human stem cell models can recapitulate Group 3 MB and serve as platforms for preclinical drug testing.

Human stem cell models have also been instrumental in exploring SHH MB development and progression. iPSC-derived NES cells overexpressing *MYCN* generated tumors *in vivo* that were more histologically and molecularly consistent with SHH medulloblastoma than previous models (Huang et al., 2019). NES cells derived from a Gorlin syndrome patient harboring a *PTCH1* mutation were engineered with *DDX3X*, *GSE1*, or *KDM3B* mutations, accelerating tumor formation while retaining a neural progenitor state and the capacity to differentiate toward the granule neuron lineage, the proposed cell of origin for SHH MB (Huang et al., 2019). This cell line was used in subsequent xenotransplantation studies which demonstrated that “tumor-isolated” NES cells taken from parental mice were able to create faster growing tumors when implanted into secondary mice (Huang et al., 2019; Susanto et al., 2020). These secondary tumors also showed reduced dependence on exogenous growth factors and increased neurosphere formation while maintaining SHH signaling (Susanto et al., 2020). The importance of SHH signaling in MB tumorigenesis has been further emphasized by Ikemoto et al. through implanting iPSCs derived from Gorlin patients into mice (Ikemoto et al., 2020). Teratomas that formed *in vivo* from this Gorlin cell line displayed MB-like regions not present in the teratomas formed from wildtype iPSCs (Ikemoto et al., 2020). Further, additional *PTCH1* mutations were observed in these MB-like regions but not in the cartilaginous sections (Čančer et al., 2019). Comparative studies between iPSC- versus ESC-derived NES cells showed that both generate SHH medulloblastoma-like tumors following *MYCN* amplification, but the iPSC-based models produce more aggressive and metastatic tumors (Čančer et al., 2019). These studies not only highlight that human stem cell models can be used to study tumor initiation and evolution in SHH MB, but also that formation of these tumors rely on certain cell types and developmental states just as much as they do on SHH pathway mutations.

iPSC-derived cerebellar organoids have also been created to better recapitulate MB pathophysiology. *PTCH1*-mutant organoids showed expression-dependent effects of SHH pathway disruption, with *PTCH1* homozygous organoids exhibiting impaired differentiation and cerebellar regional identity not seen in the heterozygous model (van Essen et al., 2024). *PTCH1*+/− organoids displayed preneoplastic features with transcriptional profiles closely resembling SHH medulloblastoma (van Essen et al., 2024). Group 3 MB has also been modeled in cerebellar organoids with c-MYC overexpression in combination with GFI1 or OTX2 (Ballabio et al., 2020). Both types of mutations resulted in impaired differentiation and expanded cerebellar progenitor populations, however, the *c-MYC+GFI1* line resulted in tumors *in vivo* that were classified as higher risk (Ballabio et al., 2020). These studies demonstrate that human stem cell-derived organoids provide a powerful platform to study oncogenic mechanisms that alter cerebellar development leading to MB initiation.

Across medulloblastoma subtypes, human ESC- and iPSC-based approaches have led modeling of MB by enabling precise genetic manipulations and their subsequent developmental contexts within early human neural development. These models have clarified key initiating mutations, defined cooperating mutations, revealed mechanisms of tumor evolution, and provided platforms for therapeutic testing in medulloblastoma.

### Ependymoma

Ependymomas are glial-derived tumors originating from the ependymal cell lineage that present significant therapeutic challenges, particularly in pediatric patients, due to their molecular heterogeneity and risk of recurrence (Jünger et al., 2021). These tumors can occur within the brain and spinal cord, and each subtype is associated with different known molecular drivers of tumorigenesis (d’Amati et al., 2024). There have been a paucity of human stem cell-based approaches due to the lack of iPSC protocols for ependymal lineage differentiation and poorly understood origins of these tumors (Taylor et al., 2005; Poppleton and Gilbertson, 2007; Gojo et al., 2020).

Many approaches to model ependymoma have relied heavily on primary tumor-derived 2D cultures and neurospheres. Previous work established posterior fossa ependymoma cell lines that retain tumorigenicity *in vivo* despite low expression of canonical NSC makers CD133 and nestin, suggesting limited stem-like populations in bulk cultures (Pavon et al., 2018). In contrast, neurosphere-based studies have demonstrated stem-like properties and formed tumors in mice that recapitulate patient histopathology (Yu et al., 2010; Milde et al., 2011; Hussein et al., 2011; Servidei et al., 2012; Meco et al., 2014). Interestingly, several studies have reported that these tumors are able to reform when serially transplanted into other mice, and these secondary transplant tumors show resistance to some inhibitors compared to primary xenografts, indicating a more aggressive phenotype that may be relevant in studying recurrent tumors (Milde et al., 2011; Hussein et al., 2011). More recently, Taylor et al. showed that hypoxic conditions may be necessary to maintain ependymoma cells in 2D culture due to toxic effects of ambient oxygen in the cell (Michealraj et al., 2020). These studies show that CSC-based neurosphere models provide unique insights into tumorigenesis and potential therapies, however, they are not sufficient to determine early events that lead to tumorigenesis.

Advances in 3D culture systems have further improved the physiological relevance of ependymoma models. A silk-protein scaffold-based 3D platform co-culturing posterior fossa ependymoma with human endothelial cells was developed which displayed microvascular rosette-like structures and maintained nestin-positive CSC populations (Tang-Schomer et al., 2023). VEGF supplementation improved tumor cell growth and preserved tumor gene signatures, suggesting a role in ependymoma formation *in vitro* (Tang-Schomer et al., 2023). Similarly, posterior fossa ependymoma cells cultured in 3D preserved cilia-related gene expression characteristic of primary tumors that were lost when cultured as a monolayer (Amani et al., 2017). Both these studies emphasize the potential importance of microenvironmental cues for maintaining key transcriptional programs in ependymoma.

More recently, an ependymoma tumor-organoid model was created by co-culturing patient tumor explants with iPSC-derived organoids (Peng et al., 2025). This model preserved histopathological hallmarks, including perivascular pseudorosettes and high tumor cell density, and retained molecular subtypes seen *in situ* (Peng et al., 2025). Although not performed on the ependymoma line, single-cell RNA sequencing of other tumor-organoids created in this study showed that 3D organoid cultures maintained cellular heterogeneity and tumor-specific transcriptional programs, suggesting that these platforms capture many aspects of tumors *in vivo* (Peng et al., 2025).

Despite these advances, most available models rely on CSCs directly derived from patient tissue or engineered neural progenitors, and many models only represent intracranial ependymomas. This gap presents a critical opportunity for generating developmentally relevant stem cell systems that can complement xenograft and tumor-organoid models for all ependymomas and provide a better understanding of the cell-of-origin of ependymoma.

### Atypical teratoid/rhabdoid tumors

Atypical teratoid/rhabdoid tumors (ATRTs) are highly malignant pediatric brain tumors that are primarily driven by *SMARCB1* inactivation and typically arise in younger patients (d’Amati et al., 2024). Progress in understanding ATRT pathophysiology has been limited by the lack of models that capture the origin and development of this disease. However, recent human stem cell-based approaches have been developed to model these processes and identify therapeutic targets.

Terada et al. engineered human iPSCs with *SMARCB1* and *TP53* mutations to interrogate how developmental timing influences ATRT tumorigenesis (Terada et al., 2019). *SMARCB1* loss impaired neuronal differentiation, whereas *TP53* loss enhanced proliferation (Terada et al., 2019). When implanted into mice, mutant iPSCs formed tumors that had histopathological and transcriptomic features similar to ATRTs (Terada et al., 2019). However, when these cells were differentiated to neural progenitors prior to implantation, the tumors still had multilayered rosettes and medulloblastoma-like features but lacked rhabdoid morphology (Terada et al., 2019). Reinduction of pluripotency in the differentiated mutant cells restored their ability to form ATRT-like tumors, suggesting that ATRT initiation may require pluripotent-like cells early in development (Terada et al., 2019). Similarly, Hue et al. also generated *SMARCB1* and *TP53* double-knockout iPSCs and differentiated them into 3D spheroids that retained pluripotency markers characteristic of ATRT (Hua et al., 2023). Drug screening was performed on this model system, identifying targets for further preclinical testing (Hua et al., 2023). A separate study developed an inducible *SMARCB1* knockdown in human iPSCs to assess the consequences of gene loss at distinct developmental stages (Parisian et al., 2020). *SMARCB1* depletion impaired proliferation and reduced cell viability in pluripotent cells, whereas neural progenitors tolerated its loss. *SMARCB1* knockdown in neural progenitors also affected the function of the BAF complex, which is seen in tumor-derived ATRT cell lines (Parisian et al., 2020). In both 2D and 3D cultures, *SMARCB1* loss disrupted differentiation within the neuron-committed lineage but not the NES or RG lineages (Parisian et al., 2020). Transcriptomic profiling revealed minimal overlap between *SMARCB1*-deficient iPSCs and neural progenitors with the neural progenitors most closely resembling the SHH subgroup of ATRT (Parisian et al., 2020). Together, these studies suggest that SMARCB1 loss results in tumorigenesis in less differentiated states, and highlights how iPSC-based models may provide mechanistic insights that are difficult to capture in tumor-derived or animal models.

### Other pediatric brain tumors

Other less common pediatric brain tumors have primarily been modeled using tumor-derived cell lines and animal systems with relatively little progress toward human stem cell-based platforms (Table 1). Expanding human iPSC- and ESC-derived models for these tumors will provide critical insights into tumor initiation, cell-of-origin, and human-specific disease mechanisms.

Tumors of the sellar region are a prime example of an area of future research. While iPSC- and tumor-derived models for pituitary adenomas, a primarily adult pathology, have been successfully established, no human-stem cell based models currently exist for craniopharyngioma (Mallick et al., 2023; Chakrabarti et al., 2022; Cui et al., 2025). Craniopharyngiomas, particularly the adamantinomatous subtype that predominate pediatric pituitary lesions, lack effective medical therapies (de Pires Oliveira Neto et al., 2025). Transgenic mouse models have provided important insights but may not fully recapitulate all the features of craniopharyngioma (Li et al., 2024; Apps and Martinez-Barbera, 2017). Engineering disease-relevant mutations into iPSCs followed by pituitary lineage differentiation as demonstrated by Mallick et al. (2023) could enable more faithful modeling of tumor development and set up a system for drug screening to identify potential therapeutic targets (Chakrabarti et al., 2022).

A similar reliance on animal models is seen in pineal gland tumors, including pineoblastomas, pineocytomas, and papillary tumors of the pineal region. Pineoblastoma, the most aggressive subtype, has been modeled using a GEMM and xenograft models (Chung et al., 2020; Kwak et al., 2016; Brabetz et al., 2018; Jiang et al., 2025). These have recapitulated some of the patient-specific markers, and have enabled mechanistic studies and therapeutic screening. However, the other pineal tumors remain largely unmodeled, and no human iPSC differentiation protocols exist to generate cells from the pineal lineage. This limitation reflects an incomplete understanding of pineal gland development but also highlights a major opportunity for human stem cell-based models. The creation of human stem cell-derived pineal models would allow for exploration of tumor initiation, cell-of-origin studies, and molecular evolution. These systems could capture human developmental timing, epigenetic states, and specific genetic vulnerabilities seen in patients, including the *RB1*- or *DICER1*-driven subtypes. Furthermore, approaches that co-culture tumor cells with iPSC-derived brain organoids may be an important next step to better understand this tumor in a human-specific context.

Embryonal tumors with multilayered rosettes (ETMRs) are another highly aggressive pediatric brain tumor with rapid progression, poor prognosis, and therapeutic resistance (d’Amati et al., 2024; Jessa et al., 2019). Research on ETMR pathophysiology and treatment has been limited by the scarcity of tumor-derived samples and the lack of preclinical models. Recently, a tumor-organoid model was created which captured histopathological features of primary patient tumors, exhibited chemoresistance pathways, and displayed RG-pericyte differentiation (de Faria et al., 2025). However, no fully stem cell-derived ETMR models currently exist that could answer questions on driver mutations, cell-of-origin, and therapeutic targets.

Choroid plexus tumors (CPTs) are epithelial tumors arising from the choroid plexus epithelium, which produces and regulates CSF (d’Amati et al., 2024; Ogiwara et al., 2012; Thomas et al., 2021). These tumors range from benign papilloma to malignant carcinomas and are challenging to treat due to aggressive growth and CSF dissemination (Dangouloff-Ros et al., 2015; Nunes do Espirito Santo et al., 2025). Preclinical studies using GEMMs harboring *MYC* or *MYCN* mutations with *TP53* loss have provided insights into tumorigenic mechanisms and potential targets for treatment (Wang et al., 2015; Peter et al., 2024; El Nagar et al., 2018; Tong et al., 2015). Tumor-derived cell lines have also been established which have been used in drug screening and basic research (Ishiwata et al., 2005; Hesham et al., 2024). Recent multi-omic and transcriptional analyses have identified candidate drivers and dysregulated pathways in CPTs, including p53, MAPK, PI3K, and RAS pathways, providing rational for designing genetic edits in future models (Choi et al., 2024; Hill et al., 2024). Taken together, these advances point toward using existing iPSC-derived choroid plexus organoids and lineage-specific differentiation protocols as a platform to build human stem cell-based CPT models. Incorporating oncogenic drivers into these systems could enable the generation of models that recapitulate CPT development and progression. Such models would bridge the gap between mouse systems and human disease, providing physiologically relevant platforms for mechanistic studies, drug discovery, and precision medicine approaches. Moreover, they could capture critical human-specific features of choroid plexus tumorigenesis that are absent in murine models, such as epigenetic state, developmental timing, and chromosomal instability.

Primary CNS germ cell tumors (GCTs) predominantly affect children and adolescents and most frequently arise in the pineal and suprasellar regions of the brain (Fetcko and Dey, 2018; Yeo et al., 2023). Their cellular origin and mechanisms of tumorigenesis remain poorly defined, but current studies suggest that CNS GCTs originate from embryonic cells that fail to properly differentiate during early development, consistent with their expression of pluripotency-associated transcription factors: *OCT4*, *NANOG*, and *KLF4* (Yeo et al., *2023*). Like the previously discussed tumors, work on GCTs largely rely on xenograft models, which exhibit histopathological and molecular features of the original patient tumor, such as *KIT* expression, but lack the ability to model early developmental origins (Lindsay et al., 2016). Given their pluripotent phenotype, using iPSC- or ESC-derived stem cells that have yet to commit to a specific lineage may offer the best chance at creating a biologically relevant human-based model. Engineering mutations that have been observed in patient GCTs (KIT/RAS, MAPK, and PI3K pathways) into these cells could provide mechanistic insight into how disrupted developmental programs may lead to malignancy (Zhou et al., 2024). Until these models are created, alternative strategies that may help bridge this translational gap include creating tumor-organoid and co-culture systems that mimic the 3D architecture and microenvironmental context of GCTs within the brain (Heinzelmann et al., 2024; Kim et al., 2020). These hybrid models have proven useful for other rare pediatric brain tumors and could offer a complementary approach to study tumor microenvironmental interactions and therapeutic vulnerabilities in GCTs.

Collectively, the lack of human stem cell models for these tumors spotlight opportunities for future research. While animal models and tumor-derived cell lines have provided foundational knowledge into mechanisms of tumorigenesis, resistance, and potential therapies, creating more biologically relevant systems may help to define the early events that cause these tumors to develop.

## Current limitations and future directions of human stem cell-based brain tumor models

### Limitations

As discussed above, human stem cell-based models have greatly expanded the understanding of tumor initiation and early tumorigenesis, specifically within a human developmental context. Yet, their current implementation as models for all brain tumors is constrained due to technical and conceptual limitations.

A central challenge of many stem cell-derived models is that they are often created without the surrounding microenvironment (Huang et al., 2021). In patients, brain tumors arise within complex regions surrounded by neural and non-neural cell types that provide vascular support, immune surveillance, and extracellular matrix components. The absence of these features *in vitro* limits their ability to capture key biological processes such as tumor invasion, immune evasion, and treatment response. While many pediatric brain tumors are thought to originate early in development, which stem cells can model well, other tumor types emerge over longer developmental windows and may not be easily modeled in culture. Furthermore, although human stem cell models can be genetically manipulated through tools like CRISPR, they often fail to mimic all the genetic, epigenetic, and biological variability seen in primary tumor tissue. Mutations engineered in these cell lines are typically introduced at one time, whereas tumorigenesis in patients may be influenced by sequential mutations (Huang et al., 2025). Moreover, the relative lack of complexity that these models have make it difficult to recreate processes seen in patients, such as tumor evolution (Huang et al., 2025).

Practical considerations also affect stem cell models, as they can be time-consuming to generate and maintain and more common pathologies are more likely to be modeled. Another potential reason for the lack of some models is limited differentiation protocols for some cell lineages from which brain tumors arise. Moreover, human stem cell models can sometimes have issues with reproducibility due to variability that can occur between different experimental batches (Selfa Aspiroz et al., 2025). Lastly, ethical concerns surrounding ESC derivation have led to restrictions on their use in some countries, while iPSCs require careful validation to ensure they accurately model disease-relevant phenotypes without reprogramming artifacts (Selfa Aspiroz et al., 2025; Lo and Parham, 2009).

### Future directions

Importantly, many of the current limitations of human stem cell-based brain tumor models represent clear opportunities for innovation. Major strides have been made through 3D culture systems, especially those that have successfully integrated microenvironmental components, like microglia, vasculature, and extracellular matrices (Cakir et al., 2019; Cakir et al., 2022; Xu et al., 2021; Wenzel et al., 2024; Dao et al., 2024; Rittenhouse et al., 2025). These platforms have created physiologically relevant models that allow researchers to better understand cell–cell interactions, immune interactions, and tumor invasion, refining our understanding of brain tumor behavior and opening the door for potential therapeutic development. Continued refinement of 3D culturing techniques will allow for better models.

Another promising direction is adapting established differentiation protocols for brain tumor modeling. As noted previously, iPSC-derived choroid plexus organoids were originally developed to study normal tissue biology but could be readily applied to model choroid plexus carcinoma through mutations in the MYC or MYCN pathway (Pellegrini et al., 2020; Jacob et al., 2020). Similar opportunities exist for other poorly studied lineages, including ependymal cells, where development of differentiation protocols would enable modeling of unique tumor types that are currently difficult to model. These are key next steps that will greatly expand researchers’ ability to model new brain tumors.

Emerging bioengineering technologies have further improved the capabilities of stem-cell based systems. Microfluidic systems, which allow for precise control over the delivery of nutrients and signaling molecules, have been used to create novel models that recapitulate early developmental signaling and processes. Recently, Xue et al. have successfully used these systems to create 3D spinal cord and forebrain models that retain regional identity of cells throughout the nervous system (Xue et al., 2025). Applying this to brain tumor models offers an ability to control spatial patterning and developmental cues that may affect tumorigenesis. Hypoxia-based culture systems have improved the physiological relevance of tumor models by better reflecting oxygen levels within the body that are known to modulate transcriptional programs (Liu et al., 2021; Blandin et al., 2019). Bioprinting methods allow additional control over the spatial distribution of cells and provide extracellular matrices that replicate those found *in vitro* (Sharma et al., 2023; Tang et al., 2020; Schroyer et al., 2025). While limited by cost, technical complexity, and bioink materials available to create structures, bioprinting is a powerful alternative to traditional 2D and 3D cultures due to its high precision and ability to construct tissue-like structures that incorporate different cell types (Sharma et al., 2023; Tang et al., 2020; Schroyer et al., 2025; Huang et al., 2024).

## Conclusion

Human stem and progenitor cell-based models have significantly advanced the study of CNS tumor biology and provide valuable tools that are complementary to GEMMs and traditional tumor cell lines. By enabling the faithful recreation of early neurodevelopmental programs, lineage specification, and tumor histopathology in a human context, these platforms uncover biological mechanisms and disease phenotypes that may not be captured in non-human systems. Their versatility, including the capacity to generate 3D models and be co-cultured with other cell types, allows researchers to create complex spatial and temporal dynamics of tumor initiation, progression, and heterogeneity.

The advancement of stem cell-based modeling will continue to reshape how we model CNS tumors. These advances will deepen our understanding of how specific genetic events overlap with developmental events to drive tumor formation and progression, as well as accelerate the discovery and testing of targeted therapies. Ultimately, human stem cell-based models may provide platforms to test the functional consequences of genetic aberrations and opportunities to stop progression of disease.
